# Patient Harm and Institutional Avoidability of Out-of-Hours Discharge From Intensive Care: An Analysis Using Mixed Methods*

**DOI:** 10.1097/CCM.0000000000005514

**Published:** 2022-03-07

**Authors:** Sarah Vollam, Owen Gustafson, Lauren Morgan, Natalie Pattison, Hilary Thomas, Peter Watkinson

**Affiliations:** 1 Nuffield Department of Clinical Neurosciences, University of Oxford, Oxford, United Kingdom.; 2 NIHR Oxford Biomedical Research Centre, Oxford, United Kingdom.; 3 Oxford Allied Health Professions Research and Innovation Unit, Oxford University Hospitals NHS Foundation Trust, Oxford, United Kingdom.; 4 Nuffield Department of Surgical Sciences, University of Oxford, Oxford, United Kingdom.; 5 School of Health and Social Work, University of Hertfordshire, Hatfield, United Kingdom.; 6 East and North Herts NHS Trust, Stevenage, United Kingdom.; 7 Centre for Research in Public Health and Community Care, School of Health and Social Work, University of Hertfordshire, Hatfield, United Kingdom.; 8 Adult Intensive Care Unit, Oxford University Hospitals NHS Foundation Trust, Oxford, United Kingdom.

**Keywords:** after-hours care, critical care, critical care outcomes, failure to rescue, human factors and ergonomics, patient views

## Abstract

**OBJECTIVES:**

Out-of-hours discharge from ICU to the ward is associated with increased in-hospital mortality and ICU readmission. Little is known about why this occurs. We map the discharge process and describe the consequences of out-of-hours discharge to inform practice changes to reduce the impact of discharge at night.

**DESIGN:**

This study was part of the REcovery FoLlowing intensive CarE Treatment mixed methods study. We defined out-of-hours discharge as 16:00 to 07:59 hours. We undertook 20 in-depth case record reviews where in-hospital death after ICU discharge had been judged “probably avoidable” in previous retrospective structured judgment reviews, and 20 where patients survived. We conducted semistructured interviews with 55 patients, family members, and staff with experience of ICU discharge processes. These, along with a stakeholder focus group, informed ICU discharge process mapping using the human factors–based functional analysis resonance method.

**SETTING:**

Three U.K. National Health Service hospitals, chosen to represent different hospital settings.

**SUBJECTS:**

Patients discharged from ICU, their families, and staff involved in their care.

**INTERVENTIONS:**

None.

**MEASUREMENTS AND MAIN RESULTS:**

Out-of-hours discharge was common. Patients and staff described out-of-hours discharge as unsafe due to a reduction in staffing and skill mix at night. Patients discharged out-of-hours were commonly discharged prematurely, had inadequate handover, were physiologically unstable, and did not have deterioration recognized or escalated appropriately. We identified five interdependent function keys to facilitating timely ICU discharge: multidisciplinary team decision for discharge, patient prepared for discharge, bed meeting, bed manager allocation of beds, and ward bed made available.

**CONCLUSIONS:**

We identified significant limitations in out-of-hours care provision following overnight discharge from ICU. Transfer to the ward before 16:00 should be facilitated where possible. Our work highlights changes to help make day time discharge more likely. Where discharge after 16:00 is unavoidable, support systems should be implemented to ensure the safety of patients discharged from ICU at night.

The link between out-of-hours discharge to the ward from ICU (during the same hospitalization) and subsequent in-hospital mortality has long been recognized (1, 2). Our recent international meta-analysis demonstrated a strong association between discharge out-of-hours from ICU and both in-hospital mortality and readmission (3). However, to our knowledge, no evidence exists to explain why those discharged at night are at higher risk of poor in-hospital outcomes. It has been suggested that more patients may be discharged to the ward overnight with limitations in their treatment (4). However, we demonstrated a similar association with readmission to ICU, suggesting patients discharged at night remain for active treatment (3), meaning care directed to treatment and cure of a condition. This association has also been suggested to be due to premature discharge because of high occupancy, with patients being moved before they are ready to accommodate admissions (1, 5). This link may also indicate that ward care is suboptimal at night (3). It is currently unclear to what extent premature discharge, high bed occupancy, or reduced care provision at night contributes to the association between out-of-hours discharge from ICU and poor outcomes. To inform changes to address this increased risk, further knowledge is required about the process of care following out-of-hours ICU discharge (6, 7).

The work reported here formed part of the REcovery FoLlowing intensive CarE Treatment (REFLECT) project, a U.K.-based mixed methods study examining the care of patients discharged from ICU to hospital wards (8). The overall aim of this program is to develop a multicomponent intervention to reduce post-ICU in-hospital mortality. In previously published work, we found out-of-hours discharge (defined as after 16:00) to occur in nearly 70% of patients whose care we reviewed (9), and this was identified as a common problem in care delivery. As out-of-hours discharge was a strong independent theme throughout the data collected, this article reports findings from the primary REFLECT data and functional analysis resonance method (FRAM) focus group related to out-of-hours discharge.

## MATERIALS AND METHODS

### Definitions

There is no consensus on the definition of out-of-hours discharge from ICU. In international literature, start times range from 16:00 to 22:00 and end times from 05:59 to 09:00 (1, 10, 11). In the United Kingdom, the common definition of 22:00 is not based on organizational changes in care provision and may be considered arbitrary (12). In our previous systematic review, discharge after 16:00 was both commonly used to define out-of-hours and associated with increased mortality and ICU readmission (3). This definition is consistent with the change from home team to on-call medical cover around 17:00, which is the usual practice in the United Kingdom (13). Patients must arrive before this time if they are to be seen by their medical team. We therefore defined out-of-hours discharges as those occurring between 16:00 and 07:59.

Premature discharge was defined as occurring for those patients experiencing ongoing clinical problems at ICU discharge which did not respond to ward-based therapy within the first 48 hours of transfer, in line with published definitions (14).

Probably avoidable death was defined as those having a greater than 50% chance of preventability if changes had been made to the care delivered (15).

### Primary Data Collection

The REFLECT study was granted ethical approval by Wales REC 4 (reference 17/WA/0139) and registered (ISRCTN14658054). Data were collected at three NHS trusts.

#### Retrospective Case Record Review.

A retrospective case record review (RCRR) of 300 patients discharged from ICU who did not survive to hospital discharge using the structured judgment review method (15) has previously been reported (9).

#### In-Depth Reviews.

**Figure 1.**
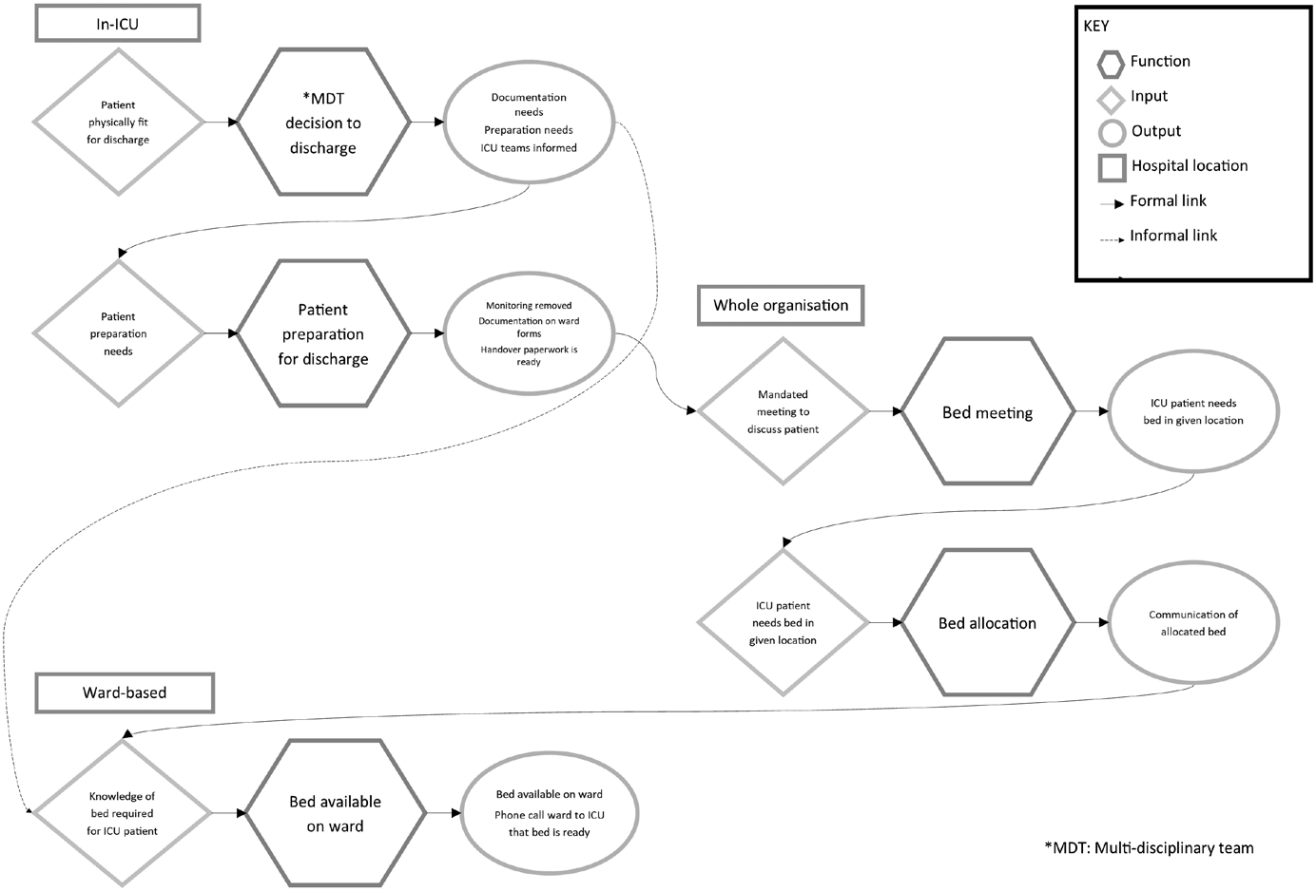
Key functions in the process of discharging a patient from ICU to the ward. MDT = multidisciplinary team.

In the RCRR, we judged 20 post-ICU deaths as “probably avoidable” (9). We undertook in-depth analysis of these 20 deaths, using an established framework (16), to find common contributory problems in care and their underlying human factors (HFs). We also analysed an equal number of survivor cases, to offer contrast with nonsurvivors (**Supplemental file 1**, **Fig. 1**, http://links.lww.com/CCM/H83). A summary of the methodology can be found in the protocol (8) and **Supplemental file 2** (http://links.lww.com/CCM/H83).

#### Qualitative Interviews.

We conducted thematic analysis (17) of semistructured interviews with 25 patients and family members and 30 staff members involved in the ICU discharge process about their experiences (Supplemental file 1, Fig. 1, http://links.lww.com/CCM/H83). Further details of the approach taken are published elsewhere (8) and summarized in Supplemental file 2 (http://links.lww.com/CCM/H83).

Findings from these in-depth analyses and interviews relating to out-of-hours discharge are presented here, including a vignette drawn from the RCRR, to offer context.

### Functional Resonance Analysis Method

The FRAM method is widely used in safety reviews in industry and increasingly in healthcare (18–20). The FRAM maps what needs to occur to ensure the intended outcome is achieved (i.e., discharge before 16:00) (20). It focuses on the functions within a process (see Table 1 for definitions). For each of these functions, the key conditions for each function to be successful are identified, defined as “input,” “output,” “precondition,” “resource,” “control,” and “time” (20). Functions link to form a process, often with the “output” of one function becoming the “input” of the next. The FRAM results in clear understanding of the steps required in a process and the conditions that have to be in place to complete these steps successfully (19, 21). We conducted a FRAM for each of the common problems in care identified, including ward-based mobilization (22).

**TABLE 1.** Definitions of Aspects of the Functional Resonance Analysis Method Process

Functional Analysis Resonance Method Aspect	Definition	Example
Function	Activity in a process	Decision to discharge from ICU
Input	Starts the function	Patient ready for ICU discharge
Precondition	Must be satisfied before the function can start	Patient does not need vasoactive drugs only administered in ICU
Resource	Needed to carry out function	Nurse time to complete documentation
Control	Monitors or controls the function	National guideline on night-time discharge
Time	Any time constraint that affects the function	Timing of bed meeting
Output	The outcome of the function	Bed allocated to patient ready for discharge

Recreated from Clay-Williams et al (20).

This FRAM, focusing on ICU to ward discharge, was informed by the primary data from the REFLECT study and input from stakeholders. An HF scientist with experience facilitating FRAMs (L.M.) led the meeting. Key staff members (external to the study team) from the three REFLECT study sites, including an ICU consultant, an ICU follow-up practitioner with a nursing background, and a senior ICU nurse, took part. Two research team members with in-depth knowledge of the primary data and ICU- and ward-based nursing and physiotherapy clinical experience also attended. At the start of the session, an information package was presented to the group, including relevant prior research and data from the REFLECT study.

The HF scientist commenced the FRAM by reiterating the focus on the “ideal world” situation—that is, discharging a patient from ICU “in-hours” (before 16:00). Group members suggested a function in the ICU discharge process. A facilitated discussion of this function took place to identify each condition (inputs, preconditions, etc.). From this, further functions and their conditions were identified, and links between the functions developed.

The HF scientist documented the developing framework on whiteboards using sticky notes color-coded to match the framework. This was refined through discussion, with changes made to functions, lines redrawn, and further functions added. The process ended when the group was happy that all functions and associated conditions directly related to the process had been captured. The final FRAM model was transcribed from the white boards into FRAM visualizer software (https://functionalresonance.com/FMV/index.html).

## RESULTS

### Primary Data Collection

#### Higher Acuity of Illness in Out-of-Hours Discharges.

Of the 40 cases reviewed in-depth, 28 were discharged out-of-hours. Five of the six discharges assessed as “premature” occurred overnight and were followed by either death or readmission to ICU within 24 hours (Table 2). Premature discharge was judged to have contributed to four “probably avoidable” deaths. The vignette (Table 3) presents a typical case of premature discharge. Premature discharges were also described in interviews as occurring more frequently at night, due to high bed occupancy and to more often result in readmission to ICU (Table 4, quotes 1 and 2).

**TABLE 2.** Frequency of Problems in Care Delivery Identified in In-Depth Reviews, Stratified by Discharge Timing

Problem in Care, *n* (%)	Out-of-Hours Discharge (*N* = 28)	In-Hours Discharges (*N* = 12)
Premature ICU discharge	6 (21)	1 (8)
Initial ward–based Early Warning Score high	12 (43)	2 (17)
Escalated as per protocol	2/12 (17)	2/2 (100)
Poor handover documentation	16 (57)	8 (67)
Medical review within 6 hr	7 (25)	9 (75)
Review conducted by Foundation Year 1/2^a^	4/7 (57)	3/9 (33)

a Foundation year (i.e., recently qualified) doctor.

**TABLE 3.** Vignette of Typical Case of Premature Discharge From ICU

Vignette: Patient discharged overnight with unresolved hypotension, which was not referred to in their handover documentation. High EWS on first observations on the ward, which was escalated. EWS was rechecked twice overnight and remained high, with no escalation. During review on the ward round in morning, there was minimal acknowledgment of their ongoing low blood pressure, tachycardia, pyrexia, and worsening hypoxia. There was no further medical documentation and infrequent observations with worsening hypotension throughout the day until critical care outreach team attend for a routine review in the afternoon, facilitating rapid ICU review and readmission, but the patient died on ICU within 24 hr.

EWS = Early Warning Score.

**TABLE 4.** Illustrative Quotes From Interviews With Patients, Family Members, and Staff, Grouped by Key Issues Identified

**Issue 1: Higher acuity of illness in out-of-hours discharges**	
Quote 1	“The sick patients that I’ve seen come from ICU that we’ve had to readmit to ITU have both been from out-of-hours discharges … none of the ones that we’ve had in-hours I don’t think have been readmitted whereas the out-of-hours ones tend to have a higher readmission.” *Foundation year doctor, staff interview 13, site C*
Quote 2	“A patient had come down from ITU, no-one really knew about this patient and he was low BP, he almost had a septic picture and was in renal failure massively and he shouldn’t have been on the ward … He came down from ICU out-of-hours which I think was because of pressures in ICU and so I can understand that but, yes, it was a bit of a worrying start to the morning.” *Foundation year doctor, staff interview 13, site C*
**Issue 2: Reduced patient safety at night**	
Quote 3	“… if they arrive in the afternoon after the ward round is done then them arriving is going to get lost with all of the other ward jobs. If they arrive at night they’re just not going to get seen I don’t think. I don’t think they’ll get seen [by senior medical staff] until the morning.” *Specialist registrar, staff interview 06, site B*
Quote 4	“... or say they’d been seen by junior doctors they won’t worry about things because they haven’t had that much experience to know what they need to worry about and so I just think it’s a stretched workforce and particularly out-of-hours more junior members of the team seeing patients.” *Specialist registrar, staff interview 5, site B*
Quote 5	“... but it was all quite unsettling that night … it was still a bit chaotic especially getting down there at 9 o’clock at night or whatever time it was and it was dark and there was darkness through the corridors of the hospital and a bit chaotic.” *Patient interview 7, site B*
Quote 6	“Bar clinicians, there’s just not enough doctors or advanced clinical practitioners available at night and at weekends and I think it’s a well-known problem unfortunately and often it’s a junior F1 or F2 doing ward cover who have got huge numbers of jobs to do and it’s not the team that’s looking after them, they don’t know the patient, they’re covering a lot more patients…” *Foundation year doctor, staff interview 13, site C*
**Issue 3: Continuity of information and care**	
Quote 7	“… quite often the patient doesn’t come down before 5/5.30 [pm] and that’s when our [specialist medical] team generally leaves the ward or around that time. I think it’s really important that the [medical] team are on the ward when a patient does arrive to be properly assessed and everything. I think that gives us a lot more confidence going into that period where it’s the evening and the night shift, it’s really important to have a clear plan of what the patient needs.” *Ward nurse, staff interview 07, site B*
**Issue 4: Unavoidability of out-of-hours discharges**	
Quote 8	“... but you don’t always get the bed until about 3 o’clock [pm] … and then you’re suddenly trying to rush everything and you’re running them out of the door … and you just by the way here you are and this is your new ward…” *CCOT/follow-up nurse, staff interview 2, site A*
Quote 9	“Patient: ... in the end it might have even been about half past 11 in the evening by the time I went. Interviewer: And you think that you went that late at night because they were waiting for the bed to be available? Patient: umm that’s my understanding yes obviously then every second is a chance someone else comes in that also needs that bed. You know and understand the game of phone calls that must go on between all the wards to try and work out where to shuffle people…” *Patient interview 6, site C*

For 12 of 28 patients discharged overnight, their initial ward Early Warning Score (EWS) was high (exceeding the threshold for protocolized escalation), suggesting failure to optimize prior to discharge (Table 2). EWSs are a weighted scoring system based on vital signs including heart rate, oxygen saturations, and blood pressure (23) (**Supplemental file 3**, **Table 1**, http://links.lww.com/CCM/H83, presents an example EWS). Only two of these high EWS were escalated, with between 3 and 9 hours to the next documented EWS (see vignette). In comparison, only two of 12 in-hours discharges had a high EWS on arrival, and both were escalated according to local protocol.

#### Reduced Patient Safety at Night.

Interviewed staff suggested decreased out-of-hours staffing and skill mix made night-time discharges more challenging and possibly less safe than during the day. In addition, the lack of specialist staff at night indicated a reduction in the safety net of support available compared with the day (Table 4, quotes 3 and 4). Patients described night-time discharge as frightening and unsettling, at a time when they were already vulnerable. Fewer staff on shift may have contributed to the perception of chaos described by one patient (quote 5). Out-of-hours was mostly discussed in reference to night, but there were also differences identified at weekends, particularly in the availability of clinical specialist and physiotherapy support (quote 6).

#### Continuity of Information and Care.

A total of 16 of 28 out-of-hours discharges had problems with handover documentation, similar to eight of 12 in-hours discharges (Table 2). The most frequent documentation problem for discharges out-of-hours was absence of a medical plan directing management of ongoing problems. In particular, ongoing problems associated with high EWS on arrival or premature discharge were rarely identified in the medical handover (see vignette, Table 3). In staff interviews, medical reviews were deemed important to continuity of care (Table 4, quotes 3, 6, and 7). However, in-depth reviews showed a medical review of any level within 6 hours of transfer was less likely to occur for discharges out-of-hours (7/28) compared with in-hours (9/12). Where review did occur, this was commonly by the most junior members of the medical team (4/7 reviews at night were by foundation year doctors). This led to more problems with the management of high EWS and premature discharges overnight (Table 4, quotes 1, 2, and 4).

#### Unavoidability of Out-of-Hours Discharges.

Staff and patients perceived out-of-hours ICU discharge as unavoidable due to high bed occupancy and delayed patient flow through the hospital (quotes 8 and 9), with staff voicing concern about the subsequent impact on patient safety and experience (quotes 1 to 8).

Overall, care provision at night was perceived as challenging due to reduced staffing and skill mix, and high workload. Staff described this as impacting their ability to manage patients who were identified as potentially being discharged before they were ready, due to high bed occupancy. These challenges augmented the perception of vulnerability experienced by patients being transferred from ICU to the ward.

## FRAM

Five functions were identified as essential to facilitating discharge from ICU before 16:00. Figure 1 presents a simplified outline of these functions, with a more detailed representation including all elements in **Supplemental file 4**, **Table 1** (http://links.lww.com/CCM/H83).

### Function 1: Multidisciplinary Team Decision for Discharge

Confirming a patient was ready for ICU discharge was identified as a multidisciplinary team (MDT) decision. Patient-based preconditions included not requiring organ support or drugs only administered in ICU and assessed by physiotherapist as ready for discharge. This decision was usually made during morning ward rounds, which may not occur until late morning depending on the unit workload.

The timing of this decision was perceived to have consequences for ward bed identification. Where the discharge decision was made the evening before, this facilitated earlier communication with the bed manager.

### Function 2: Patient Prepared for Discharge

Function 2 was also identified as an MDT process. The key precondition identified was handover documentation prepared by nurses, doctors, and physiotherapists. Drug charts, fluid balance, and vital signs charts must also be transposed into ward-friendly formats and drugs reviewed by the ICU pharmacist. This preparation requires knowledge of the ward environment and what is deliverable on the ward.

Key resources include nursing time to complete and collate documentation, remove invasive catheters and monitoring, and liaise with the ward.

### Function 3: Bed Meeting

A hospital-wide mandated morning bed meeting is held at all three organizations, to discuss discharges and bed availability. This relies on discharge information which is dependent on ICU and ward round decision-making (function 1).

### Function 4: Bed Manager Allocation of Beds

Based on bed meeting information, staffing levels, and ICU bed needs (i.e., elective surgery and emergency department admissions), a ward bed may be allocated for the ICU patient ready for discharge.

### Function 5: Bed Made Available on the Ward

Once a bed has been allocated, ICU and ward staff negotiate a discharge time and ICU inform the ward of any special requirements such as a side room (for infection control) or equipment (e.g., feed pumps, moving and handling equipment).

This function is time dependent on discharge of the patient occupying the ward bed, in turn reliant on the ward round, discharge medication preparation, and availability and appropriateness of the discharge lounge (central hospital area for patients waiting for hospital discharge).

### Final Output: Patient Discharged From ICU Before 16:00

The final outcome relies on the timely output of all the key functions, which rely on all preconditions, resources, and inputs being in place. Although the functions are not entirely linear, delay of any activity or precondition will impact the time of discharge.

## DISCUSSION

Using data from three NHS hospitals, we outlined the consequences of discharge from ICU to the ward after 16:00. Out-of-hours discharge was common at all hospitals and linked to poorer handover and a delay to first medical review. Patients discharged at night were more likely to have a high EWS on ward arrival, indicating higher acuity, which was rarely escalated overnight. Almost all premature discharges occurred out-of-hours, and consequences for these patients were profound. Both staff and patients voiced concerns about discharge at night, identified as a time of high workload and low skill mix. The combination of higher acuity of illness, a reduction in staffing and skill mix, and poorer handover of information compounds the challenges of receiving patients from ICU overnight. To examine how ICU discharge during the day may be facilitated, the process for discharging a patient from ICU to the ward was mapped in consultation with stakeholders. Examining this process identified five key functions essential to facilitating discharge from ICU before 16:00.

### Strengths and Limitations

This study had several strengths. Data were collected from three NHS trusts, as part of a larger project, which was registered and the protocol was published (8). The three sites were selected to offer contrasting characteristics such as hospital and ICU capacity and post-ICU care provision.

As the focus of the REFLECT study incorporated the whole post-ICU ward stay, data on out-of-hours discharge were somewhat limited. Future work including contextual data on bed occupancy, patient flow, organizational stress, staffing levels, and delays to hospital discharge may offer further insight into reasons for out-of-hours discharge (24). In this work, we focused on the effects of discharge out-of-hours, rather than including weekends, as previous database work suggests that these two periods differ in their effects (25). We will consider the effects of weekend discharge in future work.

Limitations of RCRR include reliance on documentation (risking loss of data and context) and hindsight bias (15, 26). By conducting interviews alongside the RCRR, we took steps to ameliorate this risk. Furthermore, care processes related to out-of-hours discharge, including written handover, time of discharge, and EWS measurement and response, were clearly documented and therefore less likely to be affected by these limitations.

Although this work was conducted solely in the United Kingdom, our meta-analysis found no difference in the association between out-of-hours discharge and poor outcome between countries, indicating this is an international problem (3). This work is therefore likely to be relevant to countries with similar healthcare systems to the United Kingdom.

The FRAM approach differed from our planned prioritization exercise in the protocol as we identified fewer distinct problems in care than anticipated. This allowed stakeholder involvement to focus on developing our understanding of each problem. The FRAM stakeholder group was relatively small but was informed by primary data from interviews with 55 patients, family members, and staff, 300 structured judgment reviews, and 40 in-depth reviews.

### Comparison With Other Literature

Our work provides context to the association identified by our meta-analysis between out-of-hours discharge and poor outcome (3), including examining the definition of “out-of-hours.” In-line with findings from our previous RCRR, both interviewee responses and in-depth reviews demonstrated that ward provision changed significantly at around 17:00. Out-of-hours care provision was limited by reduced staff ratios, increased workload, and poorer skill mix. Our choice of 16:00 as the beginning timepoint of out-of-hours discharge was supported by our primary data which showed patients should arrive on the ward with sufficient time for their home medical team to review and address any initial problems before care provision changed. Defining out-of-hours discharge from ICU between 16:00 (10, 27) and 18:00 (2, 4, 28, 29) is common in international literature.

The high frequency of “problems in care” occurring out-of-hours identified by in-depth reviews suggests significant failure of care provision overnight. Combining this with interview data offered contextual information underlying these problems. Patients discharged out-of-hours rarely received a medical review on arrival. When they did, this was usually by the most junior doctors. This suggests lack of experience may have contributed to poor outcome out-of-hours. A before-and-after study of a “Hospital At Night” multidisciplinary deterioration response initiative (30) found an increase in senior medical review may improve patient outcomes. Infrequent observations despite high EWS at night have previously been reported and attributed to high workload or limitations in clinical judgment due to inexperience of nursing and medical staff (31–33).

Interviewed staff perceived out-of-hours discharge as inevitable due to the need to create beds for incoming patients. This was supported by the high proportion of discharges occurring after 16:00 and the complexity identified in the FRAM. In-depth reviews identified several overnight premature discharges experiencing significant ongoing medical problems. Both out-of-hours and premature discharge from ICU have been identified as indicators of ICU capacity strain (34), with negative consequences for patients (35–38).

The FRAM illuminated structural issues in processes of care, amenable to changes. We identified five functions where delay would increase the risk of failure to reach the end goal of discharge before 16:00. Timing of the ICU discharge decision (function 1) relies on the timing of the morning ward round, which often occurs after the bed meeting (function 3). Making the discharge decision (function 1) the evening before ICU discharge facilitates timely information flow to the bed meeting (function 3) and therefore earlier ward bed allocation (function 4). It also gives more time to prepare the ICU patient for discharge (function 2). Finally, it allows longer for the ward to prepare a bed for the incoming patient (function 5), including facilitating discharge of ward patients to create capacity and sourcing specialist equipment. The only function where timing is immovable is the bed meeting (function 3), as it is embedded in organizational practice. Process changes need to accommodate these established practices to succeed, and the impact of delays should be acknowledged and planned for.

Although the FRAM identified strategies to support discharge before 16:00, both this work and previous literature have shown it is likely some out-of-hours discharges will be unavoidable due to limited bed capacity (1, 5, 39). In a qualitative study, this was identified as an important ethical dilemma for ICU staff, emphasizing the difficulty in balancing the needs critically ill patients within limited capacity (40). Where out-of-hours discharges have to occur, our work shows clinicians should ensure clear communication of any ongoing clinical problems in discharge documentation, a comprehensive medical review on ward arrival, and appropriate response to high EWS. These actions may be provided by the receiving ward team and/or supported by ICU clinicians where this is not possible.

## CONCLUSIONS

This study identified significant limitations in out-of-hours care provision for patients discharged from ICU overnight. Transfer to the ward before 16:00 should be facilitated where possible. Our work highlights four alterable functions where changes will help make day-time discharge more likely. Where discharge after 16:00 is unavoidable, clear acknowledgment of ongoing clinical problems in discharge documentation, a medical review on ward arrival and careful monitoring of EWS should be implemented to ensure the safety of patients discharged from ICU at night.

## ACKNOWLEDGMENTS

We gratefully acknowledge the patients, family members, and staff who participated in the REcovery FoLlowing intensive CarE Treatment study and the sites who supported us in the conduct of this research.

## Supplementary Material


