# Supplementary material for: Patient Harm and Institutional Avoidability of Out-of-Hours Discharge From Intensive Care: An Analysis Using Mixed Methods*
Source: Crit Care Med. 2022 Mar 7;50(7):1083–92. doi: 10.1097/CCM.0000000000005514 (PMC9197137; doi:10.1097/CCM.0000000000005514)
Supplement: Supplementary file 1 [file ccm-50-1083-s001.docx]

**Patient harm and institutional avoidability of out-of-hours discharge from intensive care: An analysis using mixed methods**

**Supplemental file 1**

*280 cases where avoidability was judged as less than probably avoidable*

**55 semi-structured interviews**

- 30 staff members
- 25 patients and/or family members

**RCRR: 300 post-ICU non-survivors**

**20 survivor cases**

**20 probably avoidable deaths**

Figure 1. Flowchart of participants in primary data collection

**Supplemental file 2: Further details of in-depth review approach**

**In-depth reviews**

Process:

- For each in-depth review, medical records were reviewed to generate a narrative account of care delivery.
- Each narrative account was examined in comparison with ‘theoretical problem-free care’^1^, to identify problems in care delivery.
- A short description of each problem identified was entered into an excel spreadsheet and given a ‘problem in care’ definition, derived from the narrative description.
- Each identified problem was then coded using the ‘problem in care’ framework consisting of 53 codes across eight categories of clinical care^1,2^ (table 2)
- Identified ‘problems in care’ were reviewed in the context of the narrative account and further analysed to identify any underlying human factors which may have contribute to each problem, using the Contributory Factor Classification Framework^3^

Table 1: Initial descriptive codes derived from the narrative

| **Narrative code** |
| --- |
| ***At or related to ICU discharge:*** |
| Optimisation at ICU discharge |
| Out-of-hours discharge |
| Medical review on ward transfer |
| ICU handover |
| ***Specific clinical needs or conditions:*** |
| Prognosis/complexity |
| Mobilisation |
| Chest physiotherapy |
| Nutrition provision |
| Fluid management |
| Infection management |
| ***Identification/management problems:*** |
| Monitoring |
| Monitoring (blood results) |
| Escalation |
| Management of identified problems |
| Radiological investigation |
| ***Clinical services:*** |
| Specialist input |
| Outlier for main problem |
| Outreach/follow-up services |
| Medical support and leadership |

Table 2: Problem in care codes derived from the ‘problem in care’ framework

| Problem in care category |
| --- |
| 1 Diagnosis |
| 2 Assessment |
| 3 Clinical monitoring /management |
| 4 Infection-related |
| 5 Technical problem |
| 6 Drugs and fluids |
| 7 Resuscitation |
| 8 Other |
| Total |
| Problem in care sub-category |
| 1.1 Failure to take an adequate history and/or to perform a satisfactory physical examination |
| 1.2 Failure or delay to employ indicated test. |
| 1.3 Test was incorrectly performed |
| 1.4 Test was incorrectly reported |
| 1.5 Failure or delay to receive report |
| 1.6 Failure or delay to act upon results of tests or findings. |
| 1.7 Failure to draw sensible/reasonable conclusions or make a differential diagnosis |
| 1.8 Failure or delay to get expert opinion from: |
| 1.8.1 more senior member of team |
| 1.8.2 specialist clinical team |
| 1.8.3 non-clinical specialist (e.g. radiologist) |
| 1.9 Expert opinion incorrect |
| 1.10 Other (specify) |
| 2.1 Failure to take a full clinical history |
| 2.2 Failure to examine carefully |
| 2.3 Failure to take account of co-morbidity |
| 2.4 Failure to monitor adequately |
| 2.5 Failure to record |
| 2.6 Failure to communicate to the rest of the team (clinical and multi-disciplinary) |
| 2.7 Other |
| A inadequate monitoring/management of: |
| 3.1 Abnormal vital signs (including neurological status) |
| 3.2 Problems with fluids/electrolytes including renal function |
| 3.3 Side-effects of medication |
| 3.4 Cardio-pulmonary dysfunction |
| 3.5 Damage to skin and pressure areas |
| 3.6 Adequate mobilisation |
| 3.7 Infection |
| 3.8 Poor progress in healing |
| 3.9 Changes to the patient’s general condition |
| 3.10 Other (nutrition) |
| B In what respect clinical management unsatisfactory: |
| 3.11 Failure to take note of 'routine' observations |
| 3.12 Delay in noting lab/test results |
| 3.13 Not aware of significance of lab/test results |
| 3.14 Failure to act appropriately on lab/test results |
| 3.15 Poor note-keeping |
| 3.16 Inadequate handover |
| 3.17 Lack of liaison with other staff |
| 3.18 Inadequate ‘out-of-hours’ cover/working practice |
| 3.19 Guideline/ protocol failure (either not available or not followed) |
| 3.20 Apparent failure to recognise deterioration |
| 3.21 Deterioration recognised but additional care not provided |
| 3.22 Failure to recruit help: |
| 3.22.1 Medical |
| 3.22.2 Nursing |
| 3.22.3 Ancillary |
| 3.23 Other (dehydration/malnutrition) |
| B Error in infection management: |
| A infection site 4.1-6 |
| 4.7 Failure to drain pus or remove necrotic material |
| 4.8 Failure to give appropriate antibiotics (including overuse) |
| 4.9 Failure to give appropriate physiotherapy (e.g. chest) |
| 4.10 Failure to maintain care of catheters/cannulas/drains/wounds |
| 4.11 Other |
| 5 Errors related to a procedure |
| 6.1 Error in prescription/preparation of drug/IV fluids/blood |
| 6.2 Error or accident in administering drug/IV fluids/blood |
| 6.3 Failure to monitor drug action/toxicity/fluid balance |
| n/a |

*Reproduced from Woloshynowych M, Neale G, Vincent C. Case record review of adverse events: a new approach. Qual Saf Health Care 2003;12:411–5.*

Table 3: Contributory human factors categories

| Contributory human factor category |
| --- |
| A Patient factors |
| B Task and technology factors |
| C Individual (staff) factors |
| D Team factors |
| E Work environment factors |
| F Organisation and management factors |
| G Institutional factors |
| Contributory sub-factor category |
| A:1 Condition (complexity and seriousness) |
| A:2 Language and communication |
| A:3 Personality and social factors |
| B:1 Task design and clarify of structure |
| B:2 Availability and use of protocols |
| B:3 Availability and accuracy of test results |
| B:4 Decision-making aids |
| C:1 Knowledge and skills |
| C:2 Competence |
| C:3 Physical and mental health |
| D:1 Verbal communication |
| D:2 Written communication |
| D:3 Supervision and seeking help |
| D:4 Team structure |
| E:1 Staffing levels and skill mix |
| E:2 Workload and shift patterns |
| E:3 Design, availability and maintenance of equipment |
| E:4 Administrative and managerial support |
| E:5 Physical |
| F:1 Financial resources and constraints |
| F:2 Organisational structure |
| F:3 Policy, standards and goals |
| F:4 Safety culture and priorities |
| G:1 Economic and regulatory context |
| G:2 National health service executive |
| G:3 Links with external organisations |

*Reproduced from Taylor-Adams S. Vincent, C. Systems analysis of clinical incidents: The London protocol. Clin Risk, 2004;10:211-20.*

**Interviews**

Process:

- Patients, family members and staff participants were purposively recruited at all three sites to offer a range of experiences and professional groups.
- Interviews were held either by telephone or face-to-face in a quiet room away from clinical areas.
- Two researchers with clinical ICU backgrounds undertook the interviews.
- Interviews followed a topic guide and were recorded and transcribed verbatim.
- Analysis was undertaken by SV, HT and NP, following the six steps of thematic analysis described by Braun and Clarke^4^.

**References**

1. Hogan H, Healey F, Neale G, Thomson R, Black N, Vincent C. Learning from preventable deaths: exploring case record reviewers’ narratives using change analysis. *J R Soc Med* 2014;107:365–75.
2. Woloshynowych M, Neale G, Vincent C. Case record review of adverse events: a new approach. Qual Saf Health Care 2003;12:411–5.
3. Taylor-Adams S. Vincent, C. Systems analysis of clinical incidents: The London protocol. Clin Risk, 2004;10:211-20.
4. Braun V & Clarke V. Using thematic analysis in psychology. Qual Res Psychol, 2006;3:77–101.

**Supplemental file 3: Details of Early Warning Scoring system used**

*Reproduced from Royal College of Physicians. (2012). National Early Warning Score (NEWS). London: RCP.*

Table 1: National Early Warning Score parameters

| **Physiological parameter** | **3** | **2** | **1** | **0** | **1** | **2** | **3** |
| --- | --- | --- | --- | --- | --- | --- | --- |
| **Respiration rate** | ≤8 |  | 9-11 | 12-20 |  | 21-24 | ≥25 |
| **Oxygen saturations** | ≤91 | 92-93 | 94-95 | ≥96 |  |  |  |
| **Any supplemental oxygen** |  | Yes |  | No |  |  |  |
| **Temperature** | ≤35.0 |  | 35.1-36.0 | 36.1-38.0 | 38.1-39.0 | ≥39.1 |  |
| **Systolic blood pressure** | ≤90 | 91-100 | 101-110 | 111-219 |  |  | ≥220 |
| **Heart rate** | ≤40 |  | 41-50 | 51-90 | 91-110 | 111-130 | ≥131 |
| **Level of consciousness** |  |  |  | Alert |  |  | Voice, pain or unconscious |

Table 2: NEWS thresholds

| NEW score | Clinical risk |
| --- | --- |
| 0 | Low |
| 1-4 |  |
| Individual parameter score of 3 | Medium* |
| Aggregate 5-6 |  |
| Aggregate 7 or more | High |

*Point of first protocolised escalation action

**Supplemental file 4**

Table 1. Extended detail of the identified components of the ICU discharge FRAM

| **Function: MDT decision to discharge** | |
| --- | --- |
| Input: | Patient physically fit for discharge |
| Pre-conditions: | MDT knowledge of the patient  Patient does not require organ support, drugs or other therapy not deliverable on the ward  Physiotherapy assessed as fit for discharge |
| Resources: | Full MDT team present |
| Controls: | Guidelines for drugs and other therapy not delivered on the wards |
| Time: | Decision usually made on ward round |
| Output: | Documentation requirements  Patient preparation requirements  Communication to teams re: decision |
| **Function: Patient preparation for discharge** | |
| Input: | Patient preparation requirements |
| Pre-conditions: | Handover documentation prepared by MDT  Medication prescribed in ward-friendly administration route |
| Resources: | ICU workload capacity  Knowledge of what can be delivered on the ward  Knowledge of how to prepare a patient for discharge |
| Controls: | Local ICU discharge policies  NICE Clinical Guidelines 83 |
| Time: | May be down-prioritised for other tasks in ICU |
| Output: | Monitoring removed  Documentation transcribed to ward forms  Handover paperwork ready |
| **Function: Bed meeting** | |
| Input: | Organisation-mandated meeting to discuss patient flow |
| Pre-conditions: | Knowledge of beds needed in ICU for planned and emergency admissions  Knowledge of discharges from the ward  Knowledge of planned elective surgical activities |
| Resources: | All senior nurses with bed management responsibility |
| Controls: |  |
| Time: | Mandated meeting time |
| Output: | ICU patient needs bed in given location |
| **Function: Bed allocation** | |
| Input: | ICU patient needs bed in given location |
| Pre-conditions: | Knowledge of the discharging patients clinical needs |
| Resources: | Bed manager time |
| Controls: | Local bed allocation policies |
| Time: |  |
| Output: | Communication of available bed |
| **Function: Bed available on ward** | |
| Input: | Knowledge of bed required for patient  Knowledge of patient needs |
| Pre-conditions: | Beds being available on ward |
| Resources: | Appropriate equipment available  Appropriate staffing to receive patient |
| Controls: | Local hospital discharge policies |
| Time: | Reliant on all other functions  Follows bed meeting  Bed preparation may be down-prioritised for other ward tasks  Relies of discharge of patient currently in the bed |
| Output: | Bed ready on ward  Phone call to ICU that bed is ready |
